# Community Detection in Large-Scale Bipartite Biological Networks

**DOI:** 10.3389/fgene.2021.649440

**Published:** 2021-04-21

**Authors:** Genís Calderer, Marieke L. Kuijjer

**Affiliations:** ^1^Centre for Molecular Medicine Norway, University of Oslo, Oslo, Norway; ^2^Department of Pathology, Leiden University Medical Center, Leiden, Netherlands

**Keywords:** networks, genomic networks, community detection algorithms, community detection analysis, genomic data analysis, network analysis, biological network analysis, biological network clustering

## Abstract

Networks are useful tools to represent and analyze interactions on a large, or genome-wide scale and have therefore been widely used in biology. Many biological networks—such as those that represent regulatory interactions, drug-gene, or gene-disease associations—are of a bipartite nature, meaning they consist of two different types of nodes, with connections only forming between the different node sets. Analysis of such networks requires methodologies that are specifically designed to handle their bipartite nature. Community structure detection is a method used to identify clusters of nodes in a network. This approach is especially helpful in large-scale biological network analysis, as it can find structure in networks that often resemble a “hairball” of interactions in visualizations. Often, the communities identified in biological networks are enriched for specific biological processes and thus allow one to assign drugs, regulatory molecules, or diseases to such processes. In addition, comparison of community structures between different biological conditions can help to identify how network rewiring may lead to tissue development or disease, for example. In this mini review, we give a theoretical basis of different methods that can be applied to detect communities in bipartite biological networks. We introduce and discuss different scores that can be used to assess the quality of these community structures. We then apply a wide range of methods to a drug-gene interaction network to highlight the strengths and weaknesses of these methods in their application to large-scale, bipartite biological networks.

## 1. Introduction

Many processes in biology are linked through complex patterns of physical and functional interactions, which can be represented in large-scale, genome-wide biological networks. Analysis of these networks can help our understanding of biology and medicine (Barabási et al., 2011). For example, a recent analysis of protein-protein interaction networks has helped to map cellular organization and genome function (Luck et al., 2020). Analysis of gene regulatory (Sonawane et al., 2017) and expression quantitative trait (eQTL) networks—where Single Nucleotide Polymorphisms (SNP) are connected to gene expression levels based on the strength of their association (Platig et al., 2016; Fagny et al., 2017)—have helped to highlight potential disease associations of genes and SNPs.

Most of the literature on genome-wide biological network analysis has focused on unipartite networks—networks with one type of node, where interactions can in principle form between all nodes. Examples of such networks are those that represent protein-protein interactions or gene-gene co-expression. However, many types of biological networks are naturally bipartite, meaning that there are two disjoint types of nodes, and interactions can only form between the different node types. Examples of genome-wide bipartite networks are gene regulatory networks (Emmert-Streib et al., 2014)—which include transcriptional, post-transcriptional, and post-translational regulatory networks (Koch, 2016; Statello et al., 2020; Guo and Amir, 2021)—eQTL networks, networks comprising gene-pathway associations (He et al., 2014), networks representing gene-disease (Goh et al., 2007; Halu et al., 2019) or non-coding RNA (ncRNA)-disease associations (Sumathipala et al., 2019), or drug-target interaction networks (Yildirim et al., 2007) (see Pavlopoulos et al., 2018 for an extensive overview of different types of bipartite biological networks).

**Figure 1:**
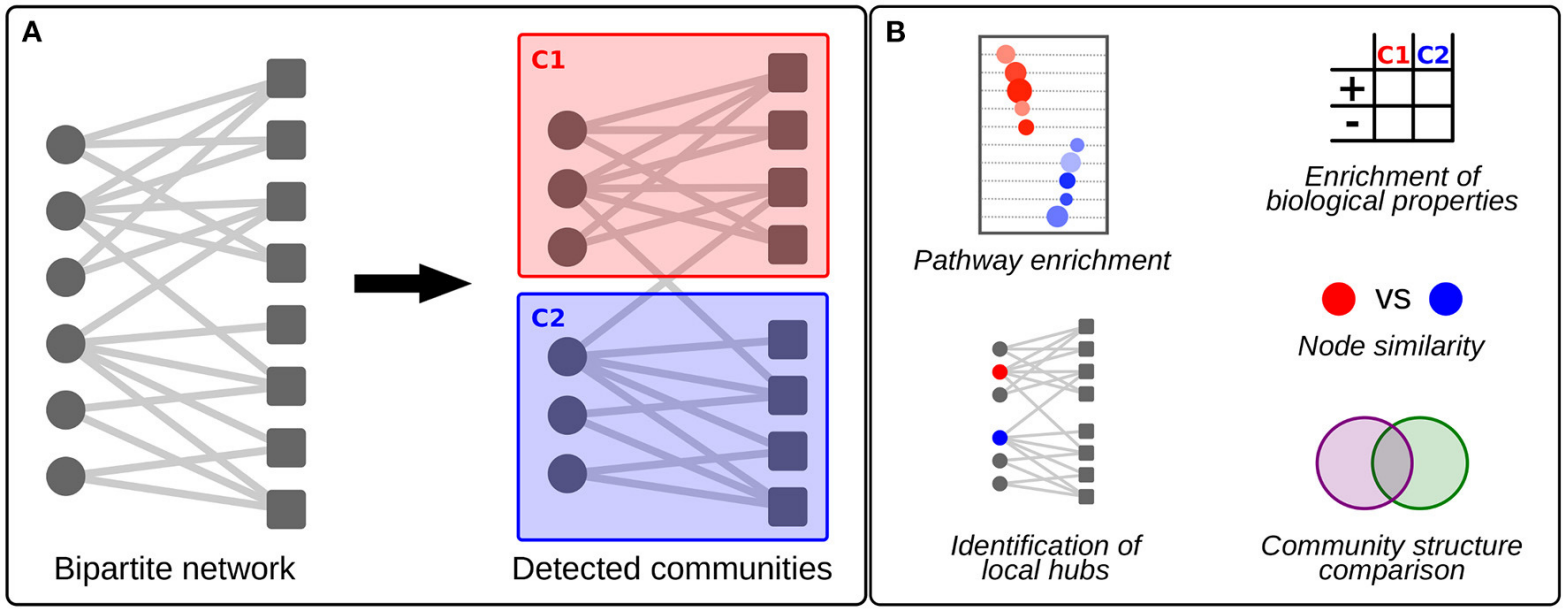
Schematic visualization of bipartite community detection and its applications to large-scale biological networks. **(A)** An example of two communities (C1 and C2) detected in a bipartite network. **(B)** Possible applications of bipartite community detection in the analysis of large-scale biological networks. This includes pathway enrichment in communities, enrichment analysis of other biological properties by testing against external data, identification of “local hub” genes that are central to their community, node similarity detection, and community structure comparison between, for example,networks modeled on disease and control samples.

Community detection is an approach to identify so-called “communities” or “modules”—sets of nodes that are densely connected internally (Newman, 2006). Community detection helps to define the higher-order structure of biological networks and allows researchers to extract and interpret biological signals (Pellegrini, 2019). For instance, in a network representing drug-gene associations, which we use as an example network in this mini review, one can apply community detection to identify groups of drugs that affect similar biological processes, thereby capturing potential new treatment strategies for patients who experience adverse effects to a specific drug. In eQTL networks, communities are often enriched for specific biological functions. SNPs in the center of these communities are enriched for regulatory elements and associated with disease phenotypes (Fagny et al., 2017). In regulatory networks,—which are often bipartite in nature, representing regulatory molecules and their targets as different types of nodes—community detection may help improve our understanding of the functions of specific regulatory molecules, as it places similar regulatory molecules in the context of their neighborhoods of targets (Sonawane et al., 2017). Community detection is particularly helpful in increasing our understanding of the biological processes that are targeted by relatively understudied regulatory molecules, for which specific functions are often unknown. These include, for example, ncRNAs (Kuijjer et al., 2020) or regulatory molecules that are not evolutionarily conserved. For a schematic overview of community detection in large-scale bipartite biological networks and their applications, please refer to Figure 1.

In this mini review, we discuss different community detection methods that can be applied to identify modules in large-scale bipartite biological networks. We start by giving a theoretical basis of bipartite networks and their community structures in general. We then discuss so-called “modularity” scores, which can be used to assess community structure quality. We show how calculating these modularity scores on bipartite networks differs from calculating them on unipartite networks. We then describe five widely used strategies for community detection that were specifically designed to be applied to bipartite networks. Finally, we assess the performance of these methods on a large-scale, near genome-wide, gene-drug interaction network and discuss the feasibility of applying these methods to genome-wide networks. We hope this overview will help shed light on the challenges with community detection in genome-wide networks in general, as well as on the advantages and disadvantages of applying some of the most widely-used community detection methods to large-scale bipartite genomic networks.

## 2. Problem Definition

We will first discuss the theoretical basis of some of the most widely used community detection methods that can be applied to networks in general (Diestel, 2005). We note that most of these methods were not initially designed for or tested on biological networks. However, they can be applied to biological networks and have been widely used in their analysis. We start by defining what a network is and, in particular, what a bipartite network represents. We also introduce the notation that we will use in the rest of this mini review.

** Definition 1**. A weighted network *G* = (*V, E*, ω) is a triple— a set of three elements—where *V* is a set of nodes, *E* is a set of edges between nodes in *V*, and ω is a function that assigns each edge *e*∈*E* a weight. We denote *n* the number of nodes and $m=\underset{e\in E}{\sum }\omega \left(e\right)$ the sum of edge weights. If a network is unweighted, ω = 1 and *m* is equal to the total number of edges. A network is said to be bipartite if *V* can be partitioned into two sets, *V*_1_, *V*_2_, such that every edge *e*∈*E* is connected to a node in *V*_1_ and to a node in *V*_2_. From now on, we will use the term *G* = (*V*_1_ ∪ *V*_2_, *E*, ω) to indicate a bipartite weighted network, unless otherwise stated.

For a unipartite network, the definition of a “community” is easy and intuitive: it is a set of nodes that are more connected within the same set compared to the rest of the network (Girvan and Newman, 2002). Given a bipartite network *G*, the problem of finding bipartite communities is more complex. We say that a *community structure* on *G* is a partition of $V_{1}=\cup_{i=1}^{l}C_{i}$ and $V_{2}=\cup_{j=1}^{k}D_{j}$, where *C*_*i*_ are pairwise disjoint subsets of *V*_1_ and *D*_*j*_ are pairwise disjoint subsets of *V*_2_, such that all nodes in a specific *C*_*i*_ are more connected to a particular subset of *V*_2_ than the rest of nodes in *V*_1_ are, and likewise for the partition of *V*_2_.

As we discuss below, there are several precise definitions of what it means *to be more connected* in a network. Most of these are based on comparing the network structure to a null model, where the nodes are randomly connected, respecting the degree distribution (Barber, 2007; Murata, 2009). This allows an extension to weighted networks, since the degrees can be substituted by the sum of edge weights. We can then define scores, generally called modularities, that precisely measure how “good” a community structure is, in the sense of how much more connected the nodes are within communities compared to the random model. Most community finding strategies identify communities by maximizing such scores (Lancichinetti and Fortunato, 2011).

## 3. Modularity Scores

The definition of bipartite modularity is an adapted version of the modularity for unipartite networks, which we will describe in the section below.

### 3.1. Unipartite Modularity

Let *G* = (*V, E*, ω) be a weighted unipartite network with *n* vertices and $m=\underset{e\in E}{\sum }\omega \left(e\right)$ edges and let this network be defined by its weighted adjacency matrix *A*. *A* is a matrix such that its *ij* entry is the weight of the edge that joins vertices *i* and *j*. In case of an unweighted network, ω = 1. If each node *i* is assigned to a community *g*_*i*_, we can define the modularity score (Newman, 2006) of this assignment as follows:

(1) $$\begin{array}{r} Q=\frac{1}{2m}\underset{i,j}{\sum }\left(A_{ij}-P_{ij}\right)\delta \left(g_{i},g_{j}\right), \end{array}$$

where *P* is a matrix with entries consisting of the expectation that *i* and *j* are connected in the null model, and δ is the Kronecker delta function. We denote *B* = *A*−*P* the modularity matrix.

If the set of nodes in a given community *C* are more connected within the community itself than would be expected given a random network with same degree distribution, then, for nodes *i, j* ∈ *C*, their corresponding entry, *B*_*ij*_, in the modularity matrix will be larger than zero. Per definition, *Q* ∈ [−1, 1]. When the given community assignment is not worse than a random partition of nodes, *Q* will be larger than or equal to zero. Such a community structure is said to be stronger when the modularity score *Q* is closer to 1.

### 3.2. Bipartite Modularity Scores

Extending the definition of modularity to adapt to the structure of bipartite networks is not completely straightforward and different approaches that do this exist. The most widely used methods are described below. Please note that these bipartite modularity scores were developed for general bipartite networks and can be calculated on any type of bipartite network, including large-scale bipartite biological networks. However, the performance of these scores has not been tested on large-scale biological networks and it is difficult to assess which method is the best. For an overview of how optimizing the different modularity scores might influence the detected community structure, please refer to (Xu et al., 2015).

#### 3.2.1. Guimerà's Modularity

The first approach to a define modularity score for a given community structure on bipartite networks was designed by Guimerà (Guimerà et al., 2007). Guimerà's modularity is the cumulative deviation of the number of edges between nodes that are members of the same bipartite community from the random expectation. This score only takes into account nodes that are in one of the bipartite sets. Because of this, it is not used in any of the community finding methods that we will explore below and, thus, we will not discuss it in more detail.

#### 3.2.2. Barber's Modularity

Barber's approach to defining bipartite modularity (Barber, 2007) is a direct adaptation of the unipartite version described in Equation (1). However, instead of working with the adjacency matrix, the biadjacency matrix Ã is used. The biadjacency matrix is the non-zero block matrix in the adjacency matrix, if we order nodes first in *V*_1_ and then in *V*_2_. The bimodularity matrix is defined as $\tilde{B}=Ã-\tilde{P}$, with $\tilde{P}$ being a matrix of expectations corresponding to a null model where nodes are randomly connected, respecting the bipartite structure and degree distribution. This results in a modularity score for assigning nodes *i* ∈ *V*_1_ to communities *g*_*i*_ and nodes *j* ∈ *V*_2_ to communities *h*_*j*_, which is defined as

(2) $$\begin{array}{r} Q_{B}=\frac{1}{m}\overset{p}{\underset{i=1}{\sum }}\overset{q}{\underset{j=1}{\sum }}\left({Ã}_{ij}-{\tilde{P}}_{ij}\right)\delta \left(g_{i},h_{j}\right), \end{array}$$

where *p* = |*V*_1_|, *q* = |*V*_2_|.

Barber's modularity score takes into account the two node types and the bipartite structure of the network. However, it forces a one-to-one correspondence between the partition in *V*_1_ and the partition in *V*_2_. Thus, each set has to be partitioned into the same number of communities. This is an overly restrictive condition, as it limits the number of possible communities to min(*p, q*) (Murata, 2009).

#### 3.2.3. Murata Modularity and Murata+ Modularity

Murata and Murata+ are two modularity scores that build on the previously defined ones. The Murata modularity score (Murata, 2009) was developed to overcome the restriction mentioned in the section above and thus does not force a one-to-one correspondence between the two partitions. It introduces the concept of a *co-cluster* of *C*_*i*_ ⊂ *V*_1_, which is the community on *V*_2_ that *C*_*i*_ shares the highest sum of edge weights with (or in the more intuitive, unweighted case, the largest number of edges).

Let $2M=\underset{e}{\sum }\omega \left(e\right)$ be the sum of edge weights. For communities *C* ⊂ *V*_1_ and *D* ⊂ *V*_2_, we define the normalized weight of their connection to be $e_{C,D}=e_{D,C}=\frac{1}{2M}\underset{e}{\sum }\omega \left(e\right)$, for *e* edges from *i* ∈ *C* to *j* ∈ *D*. Each community contributes to 2*M* with a weight of $a_{C}=\frac{1}{2M}\underset{D}{\sum }e_{C,D}$. Moreover, we can define the *co-cluster* of a community *C* to be the community *D*_*C*_ ⊂ *V*_2_ with the highest concentration of edges from *C*, that is *D*_*C*_ = arg max_*D*_(*e*_*C,D*_). With these definitions, Murata's modularity score for a given partition of *V*_1_ and *V*_2_ is

(3) $$\begin{array}{r} Q_{M}=\underset{C\subset V_{1}}{\sum }\left(e_{C,D_{C}}-a_{C}a_{D_{C}}\right)+\underset{D\subset V_{2}}{\sum }\left(e_{C_{D},D}-a_{C_{D}}a_{D}\right). \end{array}$$

This score pairs each community in *V*_1_ to a community in *V*_2_, its co-cluster, and computes the difference between intra-co-cluster edges and the expected edges in a randomly generated graph. This metric is less restrictive than Barber's modularity, because it assumes different community structures in each of the sets *V*_1_ and *V*_2_ that are related to one another by the co-cluster correspondences of each community in each of the sets.

In the *biLouvain* method (Pesantez-Cabrera and Kalyanaraman, 2016), which we describe in the next section, the definition of Murata's modularity is extended so that the co-cluster relationship is not necessarily symmetric. To do so, the choice of co-cluster is adapted to use the terms *a*_*C*_*a*_*D*_*C*__ and *a*_*C*_*D*__*a*_*D*_. This allows for even more flexibility, as the co-cluster *D* ⊂ *V*_2_ of a community *C* ⊂ *V*_1_ does not necessarily need to have *C* as its co-cluster. Thus, for a given partition, this new modularity score—which is called Murata+—has the same definition as in Equation 3, but the co-clusters are chosen as follows:

(4) $$\begin{array}{r} D_{C}=\underset{D}{\text{arg max}}\left(e_{C,D}-a_{C}a_{D}\right)\text{ and }C_{D}=\underset{C}{\text{arg max}}\left(e_{C,D}-a_{C}a_{D}\right). \end{array}$$

### 3.3. Resolution

Most community finding strategies rely on maximizing a modularity score (generally Barber's, see Equation 2). These approaches have been shown to retrieve true communities when applied to networks with a ground-truth community structure (Barber, 2007; Dao et al., 2017). However, there is a resolution limit when it comes to properly separating communities, which hampers community detection in large-scale networks. For unipartite networks, it was shown that communities with a number of internal edges $\leq O\left(\sqrt{m}\right)$ may not be detected (Fortunato and Barthélemy, 2007). While this problem was highlighted with unipartite modularity, this also applies to bipartite networks with Barber's modularity.

This poses a problem when it comes to working with large-scale networks, such as genomic networks; certain small, tightly-knit communities might be too small to detect. This is particularly relevant in the analysis of biological networks, as this means that general processes can still be detected, but that the subtle differences that distinguish, for example, a disease network from a control network may be below the resolution limit and thus could be left undetected. This can be adjusted [in the case of Barber's modularity (Equation 2)] by introducing a resolution parameter λ > 0, such that

(5) $$\begin{array}{r} Q_{B}=\frac{1}{m}\overset{p}{\underset{i=1}{\sum }}\overset{q}{\underset{j=1}{\sum }}\left({Ã}_{ij}-\lambda {\tilde{P}}_{ij}\right)\delta \left(g_{i},h_{j}\right). \end{array}$$

Then if λ > 1, more, but smaller communities are detected and if λ < 1, fewer, but larger communities are found.

## 4. Community Detection Strategies

Most community finding methods, both in unipartite and bipartite networks, are based on optimizing a modularity function. There are several strategies to do this in a fast and optimal manner (Newman, 2016), but there is no consensus on what method is best. However, all of these strategies are greedy—at each step the program tries to find the optimal next step. Thus, there is always the possibility to detect a local maximum instead of the global maximum, and therefore not the best structure. This can be an issue in large-scale biological network analysis, specifically if one aims to use the community structure to, for example, find similarities between drug targets in a drug-gene interaction network, or to get insights in potential regulatory functions of ncRNAs by analyzing a ncRNA-gene network.

Some of the most widely used strategies for optimizing modularity are discussed below.

### 4.1. Spectral Optimization (SO)

Spectral optimization methods are algorithms that take advantage of the structure of the various matrices (e.g. the adjacency matrix or the modularity matrix) associated to a network. The most widely used spectral optimization method for bipartite networks is Bipartite Recursively Induced Modules (BRIM) (Barber, 2007). BRIM uses the fact that, if *B* is the bimodularity matrix of a network, *R* is a community membership matrix for the nodes in *V*_1_, and *T* a community membership matrix for the nodes in *V*_2_, then the formula in Equation (2) can be written as follows:

(6) $$\begin{array}{r} Q_{B}=\frac{1}{m}\text{Tr}\left(R^{T}\tilde{B}T\right), \end{array}$$

where Tr is the trace of the matrix. Then, given an initial community structure on *V*_1_, the community assignment in *V*_2_ that maximizes modularity can be calculated. This is done recursively using the new assignment as initial community structure, until the modularity cannot increase further.

BRIM is considerably fast, because uses matrix multiplications, which are optimally implemented in several programming languages. However, it has the drawback that it strongly depends on the initial community structure assignment. In addition, it requires one to know the total number of communities beforehand. In large-scale biological networks, the number of communities is usually unknown (Sah et al., 2014; Gaiteri et al., 2015).

### 4.2. Projections and Adapted Unipartite Methods

A bipartite network can be projected onto one of its sets of nodes, for example *V*_1_. Its projection is a new unipartite network that has as nodes those in *V*_1_, and weighted edges corresponding to the number of shared neighboring nodes *i, j* ∈ *V*_1_ have. This projection retains part of the information about the topology of the network and can then be used to find a community structure using unipartite methods. Projections are often applied to large networks, where unipartite methods, such as Louvain (Blondel et al., 2008) or Leiden (Traag et al., 2019) can work very effectively. However, a drawback of projecting a network is that it will lead to a loss in resolution which, as we discuss above, is not ideal when analyzing biological networks. In addition, the relationship between a bipartite network and its projection is not one-to-one. Significantly different bipartite networks can have the same projection and, thus, could result in the same community structure. This could, for example, hamper the identification of differences between networks modeled on disease and control samples.

Some unipartite methods can be adapted to deal with bipartite networks by having a resolution/distance parameter set to two, which forces the method to compare nodes from the same bipartite set. This is a not an optimal approach, as it does not take into account the bipartite structure of the network. In large-scale bipartite biological networks, this structure is important, as we are often interested in understanding how two different types of components, such as transcription factors and their target genes, or diseases and genes, relate to one another. In addition, this approach is not valid for weighted networks, where the distance between the two sets is not uniformly two. Edges in large-scale bipartite biological networks are generally weighted as they are often based on effect sizes or probabilities. For example, in regulatory networks, one often estimates the likelihood of a transcription factor or ncRNA to regulate a target gene. eQTL networks can be built on the strength of SNP-gene associations. While these weighted networks can be transformed into unweighted networks by thresholding them on the edge weights, this approach is not ideal, as subtle changes in edges weights can drive biological differences (Lopes-Ramos et al., 2020). Therefore, methods that can only be applied to unweighted networks are generally not ideal for community structure detection in genomic biological networks.

### 4.3. Label Propagation (LP)

In label propagation (Liu and Murata, 2009b), each node is initialized in its own community. Then, for each community, the modularity that would be gained if the community were to be merged with another community is computed. Those merges that maximize modularity gain are then applied, and this process is repeated until the modularity cannot increase any further. When this point is reached, a condensation step is applied that generates a new network. In this new network, each node represents a community from the former network. The edges are interactions between the communities, which are weighted, for example, using the sum of weights from all nodes in a community to all nodes in the other. Label propagation can then again be applied to this network to find a new level of community structure. Further condensations can be applied until the modularity gain stabilizes. This is how the unipartite method Louvain works.

For bipartite networks this approach is adapted [for example in LPA (Costa and Hansen, 2014), DIRTLPAwb+ (Beckett, 2020), LP-BRIM (Liu and Murata, 2009a), biLouvain (Pesantez-Cabrera and Kalyanaraman, 2016)] to take the two different types of nodes in the modularity gain function into account.

It should be noted that these methods can have a stochastic component to solve ties in modularity gain. Therefore, it is possible that different runs of the method on the same network result in slightly different community structures. This could be a problem if one wants to compare community structures to, for example, detect phenotype-driven transitions in regulatory networks (Padi and Quackenbush, 2018), as it is difficult to distinguish differences caused by this stochastic component from those that arise due true biological differences in network structure. Also, as mentioned before, this can lead to detecting a local instead of the global maximum, and thereby not detecting the best community structure. Some algorithms, such as *DIRTLPAwb*+ run this approach several times and then keep the structure with the highest modularity. However, this comes with additional computational load, and may thus not be ideal for analysis on genome-wide networks.

### 4.4. Node Similarity (NS)

Node similarity algorithms, such as *ComSim* (Tackx et al., 2018) are different from the methods described above as they are not designed to optimize modularity. They define a similarity function between nodes, for example the number of common neighbors or the Jaccard similarity. They then use this function to find cycles in the network—so-called core communities—that have high similarity. These core communities do not contain all available nodes, as some nodes are left unassigned. To obtain a community structure that includes all nodes, these unassigned nodes are then added to the core community with which they have the highest similarity score.

### 4.5. Overlapping Community Detection

Overlapping methods for bipartite networks aim to give a covering of the bipartite sets that is not disjoint. This means that some nodes can be present in more than one community. This property makes sense in, for example, regulatory networks, because a transcription factor may regulate different biological functions that could be represented in different communities.

The main strategy for finding overlapping community structures in bipartite networks consist of finding bicliques—sets of nodes that form a complete bipartite graph—and then merging those based on a similarity function (see above). Two methods that implement this strategy for unweighted networks are BiTector (Du et al., 2008) and maxBic (Alzahrani and Horadam, 2019).

### 4.6. Limitations and Strengths of Published Methods in Their Applications to Genomic Networks

As discussed above, several methods for community detection in bipartite networks exist. In Table 1, we list the community detection algorithms described in this mini review, together with their community detection strategy (which we describe above), the modularity scores or similarity measures they maximize (objective function), whether they can be applied to weighted networks, and the programming language that these methods are available in.

**Table 1:** Community detection methods with their respective strategies of community detection, the used objective function, whether they allow for weighted networks, and their availability in different programming environments.

**Method**	**Strategy**	**Objective function**	**Weighted**	**Available**
BRIM (Barber, 2007)	SO	Bimodularity	Yes	R, Python
LP-BRIM (Liu and Murata, 2009a)	LP + SO	Murata	Yes	R
LPA (Costa and Hansen, 2014)	LP	Bimodularity	Yes	R
DIRTLPAwb+ (Beckett, 2020)	LP	Bimodularity	Yes	R
CONDOR Platig et al., 2016	LP + SO	Bimodularity	Yes	R, Python
ComSim (Tackx et al., 2018)	NS	Common neighbors, Jaccard	Yes	C++
biLouvain (Pesantez-Cabrera and Kalyanaraman, 2016)	LP + SO	Murata+	Yes	C++
biTector (Du et al., 2008)	Overlapping	–	No	Unavailable
maxBic (Alzahrani and Horadam, 2019)	Overlapping	–	No	C++ (not public)

*SO, spectral optimization; LP, label propagation; NS, node similarity*.

Bipartite biological networks all have the same basic properties—two disjoint types of nodes, with interactions only forming between the different node types. Therefore, in principle, any bipartite community detection algorithm can be applied to any type of large-scale bipartite biological network. There is no consensus on what method is best, and to our knowledge no benchmarking study has been performed to evaluate which methods are most appropriate for different types of bipartite genomic networks. However, as we also describe above, certain limitations can hamper community detection in these networks. We describe the most important limitations below.

Some community detection methods can only handle unweighted networks and thus can not be applied to all large-scale bipartite biological networks. Most biological networks can be both modeled in weighted or unweighted form. Gene-disease networks, drug-target networks, or pathway-gene networks have previously mostly been constructed and analyzed in unweighted form Goh et al. (2007), He et al. (2014), and Halu et al. (2019). However, they can also be estimated in weighted form by including, for example, information on predictions or associations in the edge weights (Sumathipala et al., 2019). While regulatory networks and eQTL networks are sometimes unweighted, they are more often based on likelihoods or associations. Weighted networks include more information and allow one to compare the strength, intensity, or capacity of interactions within a network or between different types of networks (Horvath, 2011). Thus, when possible, we recommend to use methods that can be applied to weighted networks.

The high computation load of many community detection methods is also a limitation and will influence the feasibility of applying community detection to genomic networks. This is particularly important in very large genomic networks, such as eQTL networks, which can include hundreds of thousands of SNPs in one of the node sets, and tens of thousands of genes in the other node set. For genome-wide bipartite networks with fewer nodes, such as gene-disease networks or pathway-gene networks, this may be less of a challenge. All methods we reviewed here have worst-case complexity *O*(*n*^3^), except in special cases where particular properties of the network—for example the presence of nodes in *V*_2_ that are mainly connected to a single node in *V*_1_—can be taken advantage of to reduce complexity to *O*(*n*^2^). However, this would require a specific implementation of the method for each particular network. The complexity of these methods means that they can be challenging to run on genome-wide biological networks, as we show in the example below.

In addition, as we describe in the section above, detecting communities using methods that rely on maximizing a modularity score may be hampered by the resolution limit. Again, this will be particularly relevant for very large networks, such as those based on eQTLs.

Finally, some community detection algorithms, including biTector and maxBic, the code to run the method is not publicly available. Thus, these methods may be challenging to run as the user would need to implement the code themselves or contact the authors to obtain it.

## 5. Application to a Gene-Drug Interaction Network

In general, most community detection algorithms are tested on small benchmark networks (Lancichinetti et al., 2008) and tests on large-scale bipartite genomic networks are lacking. We therefore wanted to test the performance of community detection methods on a near genome-wide network. As an example, we used a gene-drug interaction network from the The Drug Gene Interaction Database (DGIdb) (Cotto et al., 2018). We selected this network, because it is a well-known example of a large-scale biological network that is known to be modular (Pesantez-Cabrera and Kalyanaraman, 2016). This allows us to showcase the different methods retrieving, as we show below, significant communities.

### 5.1. Preparation of the Network

We downloaded the *interactions.tsv* file from DGIdb (Cotto et al., 2018) (accessed August 14, 2020). We removed all missing and duplicate data and kept only the confirmed gene-drug interactions. We built an unweighted bipartite network from these data representing the interactions between genes and drugs. Because all methods require the network to be connected, we kept the largest connected component (99% of the network in terms of nodes). This resulted in a network consisting of 22,693 interactions between 2,336 genes and 6,049 drugs.

### 5.2. Application of the Methods

**Figure 2:**
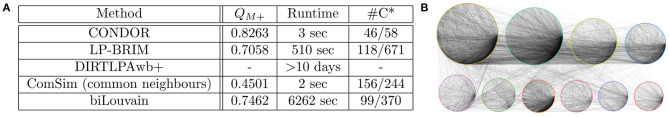
**(A)** Modularity, runtime of the method with default settings on a high-performance computing server (128 Intel Haswell cores, 1 Tb RAM), and number of communities obtained with running different community detection methods on the gene-drug interaction network. *Number of communities with more than four members/total number of communities in the gene node set. **(B)** Example “shell plot” of the ten largest communities detected in the drug-gene network using CONDOR. Communities are indicated with different colors.

We applied those community detection methods that had a functioning and available implementation to the gene-drug interaction network. As a means to consistently use the same score, we computed the Murata+ score for all of the methods. For each method, we obtained a partition of the set of genes and a partition of the set of drugs into communities. We focused on the structure in the gene node set, so that we could explore Gene Ontology enrichment and assess the significance of enriched gene sets in the different communities. Some of the communities revealed by the methods included less than four genes (see Figure 2A). We excluded these from the following analysis because they were too small to apply GO term enrichment analyses on.

The obtained modularities are shown in Figure 2A, together with the runtime and number of detected communities on the gene node set. We note that ComSim results in a significantly lower modularity score. This does not necessarily mean that the community structure is poorly defined. It is simply a result of the fact that this method does not work to optimize a modularity score. The quality of the community structure might, thus, not be captured by such scores.

An example of the ten largest communities detected with CONDOR is shown in Figure 2B. As can be seen, more edges are detected within communities compared to between different communities. However, there are also intra-community edges, indicating that community detection in large-scale networks is a complex problem.

### 5.3. Results

#### 5.3.1. Information Comparison

Because we lack a ground-truth for this network, we cannot assess the quality of results in terms of discovering a previously known community structure. However we can compare how similar the results are across the different methods. Given two community assignments on the same set of genes, we compared the information they share with the *Normalized Mutual Information* (NMI) score. This score ranges from 0 to 1, with scores closer to 1 indicating higher similarity. We computed pairwise NMIs between each of the methods. We found that the scores were similar, and contained within the [0.6077, 0.7746] range, indicating that the community assignments share a high amount of information.

#### 5.3.2. GO Enrichment

We wanted to evaluate whether the communities we discovered were enriched for specific biological processes. For each method we ran GO enrichment analysis (Klopfenstein et al., 2018) on the selected communities. All methods resulted in communities that were significantly ($p_{\text{fdr}}<10^{-8}$) enriched for biological pathways. This high level of enrichment confirms that the retrieved communities likely represent true biological information. A *t*-test concluded that there was no difference between the significance of the results for each method.

#### 5.3.3. Co-cluster Analysis

The final community structure obtained by biLouvain with Murata+ offers a relationship between communities of each of the bipartite sets. Above, we mentioned that this relationship is not necessarily one-to-one, as the co-cluster *D* ⊂ *V*_2_ of a community *C* ⊂ *V*_1_ does not necessarily need to have *C* as its co-cluster. This allows for higher flexibility when it comes to splitting particular communities in one of the sets without affecting the other. In this particular network, however, we found that the relationship was one-to-one. This might be because the network is already very modular, or the corrections in Murata+ are subtle and do not influence the final community structure strongly enough.

The co-cluster relation between communities of genes and communities of drugs is biologically significant. For example, the three largest co-clusters (based on node size) contained a co-cluster of a gene-community containing GABA genes with a drug-community that contains several benzodiazepines, which enhance the effect of GABA neurotransmitters at GABA_A_ receptors. There are several other examples of co-clusters between communities of genes of well-known pathways and communities of drugs that are known to act on those pathways (see Supplementary Table 1).

## 6. Discussion

While unipartite community detection has been widely applied to large-scale biological networks, community detection on bipartite networks and, in particular, on genome-wide bipartite networks, has been less studied. However, as many types of biological networks are bipartite, it is important to review community detection approaches that are specifically designed for such networks. Here, we reviewed several community detection strategies, discussed their strengths and weaknesses in the context of their application to genomic bipartite networks, and applied these to a near genome-wide gene-drug interaction network.

Dealing with large-scale networks is a computationally expensive task, and thus not all software packages can deal with the data in a fast manner. Although the communities detected by different methods were highly similar, the modularity scores and, in particular, their runtimes were rather different. Thus, methods that run fast could be prioritized for genomic bipartite networks. For example, as can be seen in Figure 2A, CONDOR is relatively fast on such large networks.

We would like to note that the gene-drug interaction network we included in our evaluation is indeed highly modular, and that the advantages and drawbacks of the different community detection methods might be more visible with networks with lower structure. However, there is a lack of large-scale bipartite networks with ground-truth (Peel et al., 2017) and it is very difficult to identify a large biological network that does not suffer from the resolution limit.

The Murata+ score is versatile and the communities detected by the method respect the bipartite structure of the network. However, the only method that implements it is biLouvain, which can be very slow to run on genome-wide networks. We believe that a method that uses a spectral optimizer, such as BRIM, to maximize Murata+ modularity scores would be highly useful in large-scale bipartite biological network analysis and could be a potential direction for future research.

Finally we note that, as most of the algorithms designed for bipartite community detection are focused on optimizing modularity, they may reach the resolution limit. This may render it difficult to detect communities in large-scale genomic networks and is a problem that is currently unsolved and one that warrants further investigation.

## Author Contributions

GC and MK: conceptualization, investigation, and writing—review and editing. GC: methodology, formal analysis, and writing—original draft. MK: resources, supervision, and funding acquisition.

## Conflict of Interest

The authors declare that the research was conducted in the absence of any commercial or financial relationships that could be construed as a potential conflict of interest.

## Supplementary Material

Supplementary Table 1 Co-clusters in the drug-gene network, detected using biLouvain and Murata+. Rows represent the co-clustered communities (detected on the gene node set in column 1, on the drug node set in column 2), as well as the genes and drugs present in those communities (columns 3 and 4, respectively).
